# Case studies of multi-disciplinary team management of atypical gastric cancer: challenges and lessons learned (about two cases)

**DOI:** 10.11604/pamj.2023.45.113.38579

**Published:** 2023-06-30

**Authors:** Mbang Kooffreh-Ada, Obinna Ebere Iheanacho, Victor Ikechukwu Nwagbara, Komommo Okoi Okpebri, Chukwuemeka Okechukwu Anisi, Asa Itam-Eyo, Okezie Uba-Mgbemena, Oliver Emmanuel Ali, Ofonime Benjamin Essien, Akintunde Olusijibomi Akintomide, Michael Eteng Eyong, Emmanuel Edet Effa, Ngim Ewezu Ngim

**Affiliations:** 1 Department of Internal Medicine, University of Calabar, University of Calabar Teaching Hospital, Calabar, Nigeria,; 2 Department of Haematology, University of Calabar, University of Calabar Teaching Hospital, Calabar, Nigeria,; 3 Department of Surgery, University of Calabar, University of Calabar Teaching Hospital, Calabar, Nigeria,; 4 Department of Obstetrics and Gynaecology, University of Calabar, University of Calabar Teaching Hospital, Calabar, Nigeria,; 5 Department of Orthopaedics and Traumatology, University of Calabar, University of Calabar Teaching Hospital, Calabar, Nigeria,; 6 Department of Radiology, University of Calabar, University of Calabar Teaching Hospital, Calabar, Nigeria,; 7 Department of Paediatrics, University of Calabar, University of Calabar Teaching Hospital, Calabar, Nigeria,; 8 Department of Internal Medicine, Edward Francis Small Teaching Hospital, Banjul, The Gambia

**Keywords:** Atypical, gastric cancer, management challenges, multidisciplinary team, case report

## Abstract

There is a paradigm shift towards adopting a multidisciplinary team (MDT) model in the care of cancer patients, with increasing evidence to support its effectiveness. Cancers are biologically distinct, patients present in diverse ways and require, different therapeutic approaches in their management. Patient symptoms and treatment side-effects as well as physical and psychological impact vary according to cancer location and treatment plan. The varied clinical scenarios cancer patients present further buttress the need for MDT practice in hospitals to improve the quality of patient care, in contrast to the outdated concept of holistic treatment offered by a single physician. Unlike Europe, United States of America and Australia which have implemented successful MDT cancer care programs, Nigeria is just coming on board. We present two cases of gastric cancer (seen two months apart) with atypical presentation and the role of MDT in their evaluation and management. These case studies highlight the role of MDT in the management of cancer patients in Nigeria lending credence to the urgent need to implement this model of care in our cancer patients in a bid to improve the quality of care and outcome.

## Introduction

The observed overall cancer survival rate has been proven to improve remarkably in Europe in the past four decades following the inclusion of chemotherapy, radiotherapy and/or surgery disciplines in oncology care [1]. The main objective of these MDT meetings was to develop a cohesive approach to the therapeutic plan proposed by medical oncologists, radiation oncologists and surgeons based on their clinical expertise and the evidence available to date [1]. When MDT members became aware that this approach actually improved patient care, additional specialties focused on supportive interventions were included [1]. Multidisciplinary team meetings have widely been implemented in cancer care, based on the principle that interdisciplinary case discussions lead to improved treatment recommendations based on updated and evidence-based knowledge or expert opinion[1]. The MDT meeting structure is broadly considered to improve communication, coordination and decision-making. The meetings form weekly clinical duties for most physicians, nurses, and other relevant health professionals [2]. Additionally, MDTs link clinical information from various sources, improves care processes, and adherence to clinical and up-to-date treatment recommendations, which have been documented in several cancer types [2]. Other potential benefits include shorter lead times, increased attention to patient-related perspectives, competence development, training opportunities for younger colleagues and the possibility of identifying patients eligible for clinical trials [3].

## Patient and observation

### Case 1

**Patient information**: a 28-year-old male undergraduate presented to the Accident & Emergency unit with abdominal pain of three weeks´ duration and associated abdominal swelling of two weeks´ duration. Abdominal pain was located at the periumbilical region, colicky in nature, not radiating, with no known aggravating or relieving factors. There was no history of vomiting, no diarrhoea or constipation and no jaundice. Abdominal swelling initially affected the periumbilical region and then progressed to become generalized. No renal or cardiac symptoms. He had no past medical history of note. However, there was a history of significant alcohol intake and smoking of cigarettes (including Marijuana).

**Clinical findings**: on physical examination, he was an acutely ill-looking young man in moderate painful distress. He was afebrile, anicteric, and not pale. He neither had significant peripheral lymph node enlargement, finger clubbing nor pedal oedema. His abdomen was uniformly distended, and tense with visibly distended superficial abdominal veins. There was marked tenderness especially around the umbilical region with rebound tenderness. The umbilicus was also hard and nodular. There was no organomegaly, but he had ascites. Ascitic tap done was haemorrhagic. Digital rectal examination was unremarkable. Other systemic examinations were essentially normal. An initial clinical diagnosis of acute abdomen with ruptured viscus was made, with prompt referral to the surgeons.

**Diagnostic assessment**: preliminary results of investigations revealed; full blood count - lymphopenia (19.9%) and eosinophilia (see investigation table below), erythrocyte sedimentation rate (ESR) was 27mm/hr, urea and electrolytes, urinalysis, liver function tests were essentially normal. Viral screenings were negative. An Abdominal ultrasound scan revealed mild hepatomegaly, ascites and marked probe tenderness over the umbilicus. Chest x-ray was normal while a plain abdominal x-ray done showed abnormal bowel gas distribution with multiple abnormally distended peripherally sited proximal and distal large bowel loops with multiple air fluid levels. There were no features suggestive of bowel perforation. A surgical abdomen was then excluded by the attending surgical unit. Following a review by the gastroenterology team due to persistent severe abdominal pain, swelling and bloody ascitic tap, a diagnosis of haemorrhagic pancreatitis was considered. The following additional investigations were done; ascitic fluid analysis- high serum ascitic albumin gradient (1.7g/dl), ascitic glucose was raised (5.2mmol/l, <3.5mmol), microscopy showed an inflammatory smear, eosinophils were not detected. Serum amylase was mildly elevated, whereas serum lipase was normal. Ascitic fluid geneXpert was negative for mycobacterium tuberculosis (Table 1). The patient was also unresponsive to spironolactone and frusemide. Repeated abdominal-paracentesis were carried out (with the administration of salt-poor albumin) to relieve the distension, however with rapid re-accumulation.

**Table 1:** summary of case 1 laboratory results

Chest X-ray	Ascitic fluid analysis	Tumor Markers	Umbilical nodule biopsy report	Abdominal Ultrasound scan
Normal findings	No eosinophils detected Gene Xpert- negative for Tuberculosis SAAG*= 1.7g/dl Gross Amber turbid fluid, microscopy shows pauci-cellular smear mixed inflammatory cells mainly lymphocytes and neutrophils with necrotic debris and red blood cells. Diagnosis: inflammatory smear.	Carcinoembryonic antigen = 1.5ng/ml (0.0-5.8) CA 19-9 = 29.7U/ML (0.0-37) PSA = 1.0 NG/ML (0.0-4.0) AFP = 13.0 NG/ML (0.0-10)	Suggests Metastatic Signet Ring Carcinoma	Liver enlarged (15.92cm), ascites seen with bowel loops floating within, probe tenderness over umbilicus and RIF*. Features suggestive of peritonitis.

**Liver Function Test**	**Urea & Electrolytes**	**Viral Screenings: HIV, Hbsag, Anti-HCV**	**Full Blood Count & Erythrocyte Sedimentation Rate**	**Other Ancillary Tests**
Total Bilirubin= 12 Umol/L (2-17) Conjugated Bilirubin= 6umol/L (2-7) ALT = 7 IU/L (5-40) AST=44 IU/L (5-40) ALP=70 IU/L (22-160)	Urea = 2.9 Mmol/L (2.5-4.1) Sodium= 134 Mmol/L (135-140) Potassium= 3.8 Mmol/L (3.5-5.5) Chloride=100mmol/L (96-105) Bicarbonate =25mmol/L (22-28) Creatinine=91.4umol/L(67-114)	Non- Reactive	White Cell Count= 6.1 X 103/L (4-12) Lymphocyte= 19.9 % (25-50) Monocyte= 6.9 % (2-10) Neutrophils= 63.7% (50-80) Eosinophil= 9.2% (0.0-5) Basophil= 0.3% (0-2.0) Red Blood Cell Count= 5.2 X106 PCV= 46.8 (35-55%) Platelet Count=231x103 (150-400) Erythrocyte Sedimentation Rate = 27mm/Hr	Serum Lipase= 26IU/L (<60) Serum Amylase= 94IU/L (15-90) Serum Calcium= 1.3mmol/L (<2.0) Serum Phosphate= 1.9mmol/L (<1.45)

* RIF- Right iliac fossa *Serum ascitic albumin gradient

**Figure 1:**
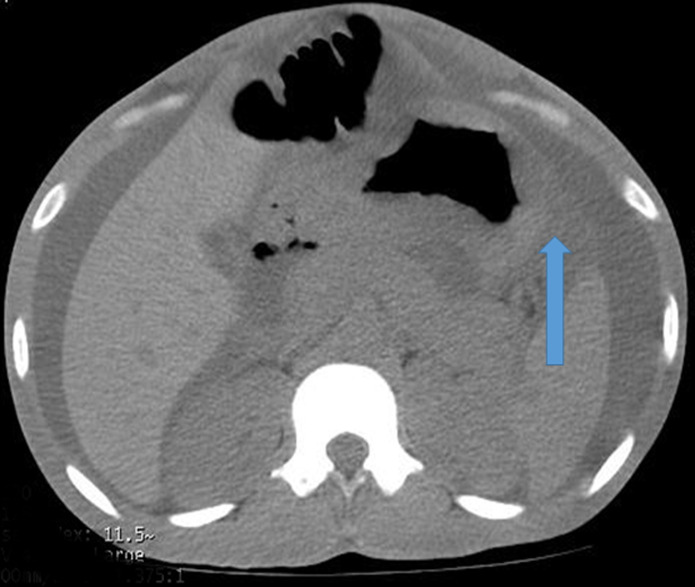
axial view of abdominal CT scan showing gastric wall thickening; differential, Linitis plastica

**Figure 2:**
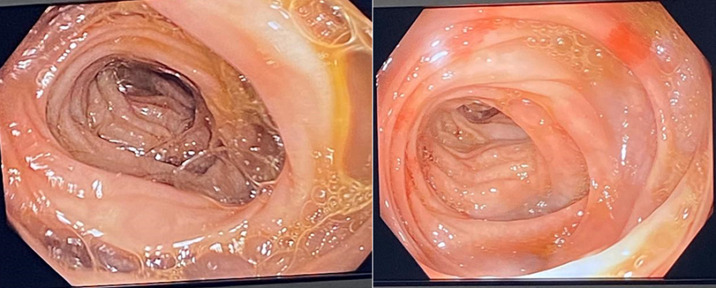
normal colonoscopy findings

**Diagnosis**: at this point, an MDT meeting and joint bedside re-evaluation of the patient was held and involved the surgeons, physicians, and radiologists. The diagnosis was further reviewed to refractory ascites secondary to a gastric malignancy (with the presence of Sister Mary Joseph´s nodule). Tumour markers (carcinoembryonic antigen, CA19-9, alpha-fetoprotein, prostate-specific antigen) were all within normal reference limits (Table 1). Histology of biopsy of the umbilical nodule was suggestive of metastatic signet ring carcinoma. Abdominal CT scan showed normal liver size, no masses seen, copious hypodense ascites, with an umbilical hyperdense mass. The Gastric wall was grossly but uniformly thickened. A differential of Linnitis Plastica was made (Figure 1). He was booked for endoscopy (upper and lower) but he was too ill to withstand the entire procedure (however colonoscopy was normal, Figure 2).

**Therapeutic interventions**: with these findings, the MDT decided to commence the patient on a trial course of cytotoxic medications (Capecitabine).

**Follow-up and outcome of interventions**: however, he passed on two weeks after this decision was taken. The bereaved family, declined an autopsy which would have been helpful to obtain a histological diagnosis.

### Case 2

**Patient information**: a 53-year-old man presented to the Accident and Emergency (A & E) unit with a one-week history of passage of melena stools and associated fainting spells. There was also a history of epigastric pain prior to onset of symptoms, however no hematemesis or hematochezia. No abdominal swelling or jaundice. However, the patient admitted a history of chronic use of non-steroidal anti-inflammatory drugs (NSAIDs) for chronic back pain (he was admitted into the A & E ward a month earlier for severe lower back pain where NSAIDs were given to alleviate the pain). Patient drinks alcohol significantly but does not smoke cigarettes. He is a known hypertensive but not diabetic.

**Clinical findings**: a general physical examination revealed a middle-aged man, obese, moderately pale, with no peripheral stigmata of chronic liver disease and no pedal edema. Systemic examination revealed an abdominal fat apron with epigastric tenderness. No organomegaly, no ascites. Digital rectal examination revealed normal prostate, and examining finger was stained with melena stools. He was hemodynamically unstable with tachycardia and orthostatic hypotension. An initial clinical diagnosis of NSAID-induced erosive gastritis (complicated by hemodynamic instability) was made. He was subsequently resuscitated (with saline infusion and blood transfusion) and given intravenous proton pump inhibitors. Upper GI endoscopy was deferred due to his ill clinical state.

**Figure 3:**
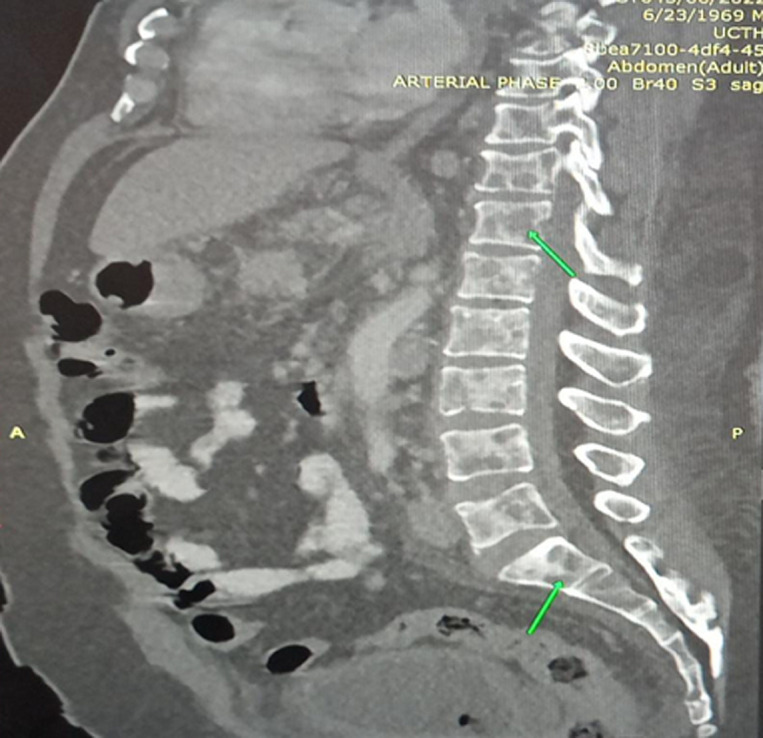
sagittal view showing diffuse lytic lesions in the lumbar vertebrae

**Figure 4:**
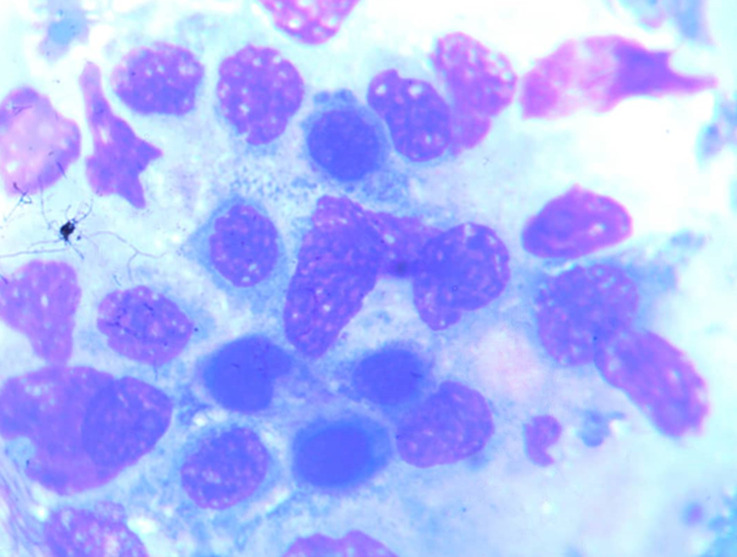
bone marrow aspiration (bma) slide showing foreign cells and numerous dying cells suggestive of marrow infiltration with increased ineffective haemopoiesis

**Figure 5:**
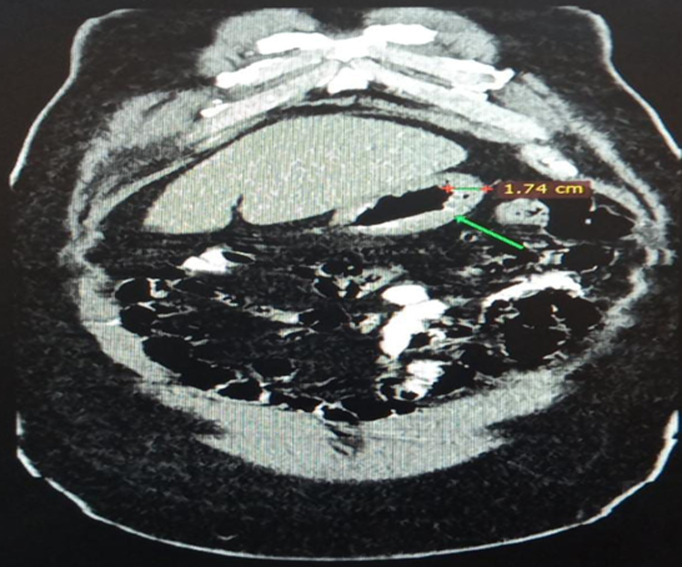
coronal view of abdominal CT scan showing gastric wall thickening

**Figure 6:**
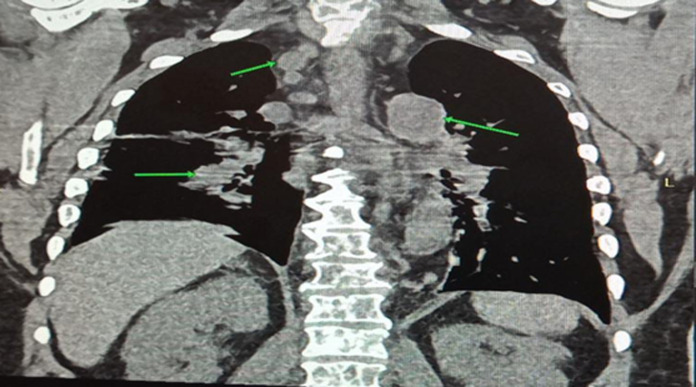
axial chest CT scan showing right para tracheal and bilateral hilar lymphadenopathy

**Diagnostic assessment**: preliminary tests showed abnormal haematological findings such as; markedly prolonged prothrombin time, deranged INR, anaemia and severe thrombocytopenia as well as leucoerythroblastic picture on peripheral blood film. These results coincided with the presentation of frank haematuria as well as desaturation in room air (with the patient requiring supplemental oxygen PRN). Other ancillary investigations such as liver function test, serum electrolytes, serum calcium, serum protein, and ESR were unremarkable (Table 2). An initial CT scan of the lumbosacral spine, however, noted widespread patchy osteolytic bone lesions within the vertebral bodies, pelvic bones and proximal femur suggesting multiple myeloma to rule out metastasis to the spine/bones (Figure 3). Prostate- specific antigen (PSA) was within the normal range. However, serum LDH was markedly raised (Table 2). Bone marrow aspiration cytology was suggestive of metastatic secondaries to the bone marrow to rule out myelodysplastic syndrome & multiple myeloma (Figure 4). A chest & abdominal CT scan revealed the florid bone involvement earlier noted as well as inhomogeneous infiltrates in the lungs and lymphadenopathy (hilar, paratracheal, mesenteric and retroperitoneal). Gastric wall thickening was also evident (Figure 5, Figure 6).

**Table 2:** summary of case 2 laboratory results

Liver function test	Urea & Electrolytes	Viral screening: HIV, HBsAg , Anti-HCV, HBV seromarkers	Full blood count + Erythrocyte sedimentation rate	Other ancillary tests
Total bilirubin= 12.8umol/L (2-17) Conjugated bilirubin = 3.4umol/L (2-7) AST= 25IU/L (5-35) ALT =22IU/L (5-45) ALP= 216.8IU/L (98-270) Total protein =5.39g/dL (6.1-8.3) Albumin= 2.14g/dL (3.8-4.4) Globulin - 3.25g/dL (2.3-3.5)	Urea = 2.9 mmol/l (2.5-4.1) Sodium=139mmol/L (135-140) Potassium=3.6mmol/l (3.5-5.5) Chloride=108mmol/l (96-105) Bicarbonate=19mmol/l (22-28) Creatinine=107.6 umol/L (67-114) eGFR=69mL/min/ 1.73m2	All Negative	White blood cell count = 9.23x103 (4-12) Lymphocytes = 37.2% (25-50) Monocytes = 7.9% (2-10) Neutrophils = 4.99% (50-80) Eosinophils= 0.5% (0.0-5) Red cell count= 3.48x106 (4.3-5.8) PCV= 25.6% (35-55) Platelet count = 41 x103 (150-400) ESR= 16mm/hr	Serum Calcium= 2.02 (2.02 - 2.60) LDH = 1303.2 U/L (240 460) HBA1c - 4.9% RBS =10mmol/L Indirect Coombs Test- Negative Retic Count - 2.4% (0.5 - 2.5) INR = 2.6 Prothrombin test : • Test =22 sec • Control=11sec
**Chest CT scan**	**Abdominal CT scan**	**Tumour Markers**	**CT scan of the spine**	**Abdominal ultrasound scan**
Mild bilateral pleural effusion. Para tracheal and bilateral hilar lymphadenopathy	Showed gastric wall thickening. and retroperitoneal lymphadenopathy	PSA = 0.4 ng/ml (0.0-4.0)	Lytic Lesions affecting L4, L5 vertebral bodies. Impression: Hematological malignancies/ Metastatic bone lesion.	Hepatomegaly with granular parenchymal echo pattern
**Bone marrow aspiration (cytology)**	**Serum protein electrophoresis**	**Microbiology work up**	**MRI lumbosacral spine**	**Endoscopy**
Suggestive of metastatic secondaries to the bone marrow to rule out myelodysplastic syndrome & multiple myeloma	Negative for multiple myeloma i.e. no monoclonal band and free light chain detected	COVID- 19 PCR - Negative Blood culture - Negative Invasive fungal infection tests- Negative	Metastatic vertebral disease with multilevel spinal cord compression - intervertebral disc herniation with thecal cauda-eqina & nerve root compression	Deferred due to the ill state of the patient

**Diagnosis and therapeutic interventions**: following an MDT meeting (physicians, haematologists and orthopaedic surgeons), the diagnosis was further reviewed to Disseminated Intravascular Coagulation (DIC) from an unknown primary cancer. Based on the outcome of the MDT meeting, he had transfusion support with fresh whole blood, fresh frozen plasma and platelet concentrate. The results of the serum protein electrophoresis and serum free light chain were not suggestive of Multiple myeloma. Bone marrow biopsy was planned but deferred as the patient was unfit for the procedure (due to the presence of bleeding diathesis). Screening tests for COVID-19 and invasive fungal infection were negative (Table 2).

**Follow-up and outcome of interventions**: three weeks into his management, he desaturated in room air less frequently, the passage of melena stools abated, the haematuria resolved. However, five days later he suddenly developed paraplegia. A magnetic resonance imaging of the lumbosacral spine revealed metastatic vertebral disease with multilevel spinal cord compression - intervertebral disc herniation with thecal cauda-equina and nerve root compression (Table 2). Based on this development, he was subsequently placed on high-dose steroid therapy. However, in accordance with the patient´s (and family´s) request, he was referred to the USA for further evaluation and care. Three weeks after arrival at a cancer hospital in the USA, a conclusive diagnosis of gastric cancer with distant metastasis was made. Unfortunately, he reportedly passed on.

**Informed consent**: it was obtained from the patients’ relatives following their demise.

## Discussion

Historically, the concept of MDT existed five decades ago in the United States and was referred to as tumour boards, multidisciplinary cancer conferences, multidisciplinary case reviews, or multidisciplinary clinics, in different health care systems [3]. The initial goal was purely academic rather than improving patient care. However, in the last two decades, there has been a paradigm shift towards community-based cancer care with improvement in patient care [3]. In a European review, the multidisciplinary approach in cancer care began in the mid-1980s when the inclusion of chemotherapy, radiotherapy and/or surgery was observed to improve overall survival [1]. With the success observed, additional specialties were included, focusing on supportive interventions [1]. The addition of these specialists further enhanced the quality of cancer care by preventing and reducing treatment side effects, which in turn improved patient adherence and compliance to therapies [1]. Worldwide, the concept of MDT is widely accepted as the “gold standard” of cancer care delivery [2]. Taberna *et al*. eloquently define MDT in cancer care as ‘the collaboration between varied specialist health care workers involved in cancer care with the all-encompassing purpose of improving the holistic management of cancer patients ’ [2]. Cancer MDTs, and MDT meetings in particular, are at the core of an increasingly complex health care system [2].

An MDT approach in the care of cancer patients in Nigeria, albeit familiar, is yet to be fully adopted. Peculiar challenges such as the dearth of trained specialists e.g. medical oncologists as well as lack of infrastructure (radiotherapy centre´s) are identifiable factors that have prevented the optimal care of cancer patients. Global data reports that gastric cancer is the third leading cause of cancer death and this is reflected in the two cases presented [4]. In Africa, gastric carcinoma has a relatively higher incidence in Nigeria and South Africa than in parts of West (French-speaking) and North Eastern Africa [4]. The Nigerian prevalence rate is reported to be between 1.64% and 4.1% [4]. Early gastric cancer is usually asymptomatic, with about half of cases having non-specific GI symptoms like dyspepsia [5]. The clinical presentation of gastric cancer in Nigeria is typically of advanced disease often complicated by gastric outlet obstruction, haematemesis or perforation [5]. However, the clinical presentation of the patients described in this case report was uncharacteristic. The aim of presenting this case report of two patients with atypical presentation of gastric cancer, was to reflect on the challenges involved in their management. The initial lack of cohesion arising from the multi-consults sent to the various sub-specialties (based on the varied clinical, laboratory and radiological presentations of the patients) further compounded the problem.

The first case of a 28-year-old male student who was initially evaluated for a surgical abdomen and then acute pancreatitis yielded inconclusive laboratory findings. However, following a MDT meeting where his case was deliberated upon by various specialists, additional investigations were recommended such as endoscopy, abdominal contrast-enhanced computed tomography scan (CT scan) and an umbilical biopsy. The patient was too frail to withstand completion of the upper and lower gastrointestinal endoscopy (gastric biopsies could not be taken). In the absence of a gastric biopsy histology report, the finding of metastatic signet ring carcinoma from the umbilical biopsy histology report and CT scan showing gastric wall thickening (Linitis Plastica) led to the working diagnosis of refractory hemorrhagic ascites secondary to gastric cancer. In one case report, the result of gastric biopsies obtained during endoscopy were inconclusive for malignancy [6]. However, the additive role of imaging (abdominal CT scan) and advanced endoscopic techniques (endoscopic ultrasound), has been found to be useful in establishing the diagnosis of Linitis Plastica [6]. The term Linitis Plastica can be used alternatively to describe stage IV gastric cancer [6]. The presentation of abdominal pain and anorexia in our index patient was similar to a report by Muraoka *et al*. [6]. They demonstrated a normal CEA but deranged CA19-9 in their patient, however, our index patient had normal tumour markers which suggests that these tumour markers may not always be reliable in the evaluation of gastric cancer. Hence, a high index of suspicion is required in the work-up of these patients. The presence of refractory haemorrhagic ascites and Sister Mary Joseph nodule in our patient suggested spread of the cancer to the peritoneum as well as the umbilicus, which is in keeping with terminal/advanced cancer [7]. The manifestation of a Sister Mary Joseph nodule is usually insidious, arising in only 1-3% of intraperitoneal malignant tumours, indicating that it is a relatively rare form of metastasis [7].

Regarding the second case, the MDT meeting also played a pivotal role in arriving at a more cohesive approach in the resultant working diagnosis of Disseminated Intravascular Coagulopathy (DIC) from a gastric malignancy. Bony metastases preceding gastrointestinal symptoms is uncommon in gastric cancer and rarely reported in literature [8]. Our index patient admitted epigastric pain, but this coincided with the use of NSAIDS for the relief of chronic low back pain (hence the initial diagnosis of possible erosive gastritis). Disseminated bony metastasis is known to have a very poor prognosis and outcome [8]. The incidence of bony metastases reportedly ranges from 1% to 45% in screening populations, suggesting that many cases are asymptomatic [8]. Bone metastasis may occur more frequently in cases with primary cancer with diffuse involvement of the stomach or a Borrmann type 4 morphology, poorly differentiated adenocarcinoma, signet ring cell carcinoma, a relatively younger age and in some cases abundant lymph node metastasis in the surrounding areas [8]. Furthermore, the cancer cells diffusely proliferate in the bone marrow and this can lead to disseminated carcinomatosis. In this situation, there is rapid proliferation that induces bone destruction as well as haematological complications such as DIC which was a prominent presentation in our index patient [8]. The exact mechanism of spread of tumour cells to the bone is unclear [8]. It has been suggested that the rich supply of blood capillaries in the gastric mucosa contributes to the early spread of cancer to the bone. An alternate non-portal route through the vertebral venous plexus has also been postulated [8]. Ben Ameur *et al*. in their case report documented similar findings (in a middle-aged female patient) as our patient [8]. The lesson learnt from the above case presentations was that physicians need to consider early MDT evaluation of cancer patients more so in complex scenarios. The rationale for introducing MDT meetings in the management of complex diseases (especially cancer), is to involve all relevant (specialist) professional groups in making clinical decisions for individual patients [2]. Core MDT expertise differs between cancer diagnosis, but usually involves surgeons, medical oncologists, radiation therapists, radiologists and pathologists. More recently, experts in nuclear medicine and molecular pathology, contact nurses, research nurses etc. have been considered [3,9].

Though both patients reported in this case study had terminal cancer, the cohesive management rendered to them by the team of specialists was very helpful. This was evident by the effective communication that enabled successful team work among the specialists. As well as, improve the interaction with the patients and their relatives. This, in particular, facilitated decision-making and planning of care for the patients. Paradoxically, the greater recruitment of multiple disciplines in cancer care, does not necessarily translate to a more effective decision-making and management approach [9]. Even when MDT cancer care has been adopted, there could be relatable problems such as; excessive caseload, low attendance at MDT meetings, poor teamwork, lack of leadership, role ambiguity, and lack of attention to the patients holistic needs [10]. Hence, factors that will positively influence the outcome of MDT meetings should be considered such as participation from qualified and effective experts, case selection, access to relevant information, discussion format and structure, leadership, health professionals´ interactions, technical equipment and administrative processes [3,9]. The perception of MDTs has been majorly positive among cancer care providers [10]. A study conducted among breast cancer health professionals in the UK, and replicated internationally showed that over 90% of respondents agreed that effective MDT care results in improved clinical decision-making, better coordinated patient care, more evidence-based treatment decisions, and improved overall quality of treatment [10].

**Limitations**: obtaining a confirmatory diagnosis (histological) of gastric cancer through gastric biopsies was greatly hindered during the work-up of the patients due to their gravely ill status. Also, the bereaved families were not keen to authorize autopsies for their relatives. Additionally, in the absence of a robust health insurance scheme in Nigeria, MDT cancer care maybe a mirage due to the high cost of out-of-pocket expenses patients encounter, while carrying out sophisticated investigations and treatment.

## Conclusion

The MDT structure is broadly considered to improve communication, coordination and decision-making among cancer care providers. This case report highlights the pivotal role of MDT in the management of cancer patients and the need to implement this care model to improve cancer morbidity and possibly mortality outcomes in Nigeria. In addition, establishing a health insurance scheme that covers cancer care is crucial in sustaining this model of care.
